# Association of serum copper (Cu) with cardiovascular mortality and all-cause mortality in a general population: a prospective cohort study

**DOI:** 10.1186/s12889-023-17018-3

**Published:** 2023-11-01

**Authors:** Xiaozhong Li, Jitao Ling, Qingwen Hu, Changchang Fang, Kaibo Mei, Yifan Wu, Jingyi Huang, Qin Ling, Yixuan Chen, Peng Yu, Xiao Liu, Juxiang Li

**Affiliations:** 1 Department of Cardiovascular Medicine, The Second Affiliated Hospital of Nanchang University, Nanchang, 330006 China; 2 Department of Endocrinology Medicine, The Second Affiliated Hospital of Nanchang University, Nanchang, China; 3 Department of Anesthesiology, The People’s Hospital of Shanggrao, Shanggrao, Jiangxi China; 4 Department of Cardiology, Sun Yat-sen Memorial Hospital of Sun Yat-sen University, Guangzhou, Guangdong China; 5 Guangdong Province Key Laboratory of Arrhythmia and Electrophysiology, Guangzhou, Guangdong China; 6 Guangzhou Key Laboratory of Molecular Mechanism and Translation in Major Cardiovascular Disease, Guangdong Provincial Key Laboratory of Malignant Tumor Epigenetics and Gene Regulation, Guangdong-Hong Kong Joint Laboratory for RNA Medicine, Guangzhou, Guangdong China

**Keywords:** Copper, Cardiovascular Diseases, NHANES

## Abstract

**Background:**

Copper (Cu) homeostasis and Cu-induced cell death are gaining recognition as crucial processes in the pathogenesis of cardiovascular disease (CVD). Circulating Cu associated with CVD and mortality is yet to be fully elucidated.

**Objective:**

This national prospective cohort study is to estimate relationship between serum Cu and the risk of CVD and all-cause mortality.

**Methods:**

This study included participants from the National Health and Nutrition Examination Survey 2011–2016. Weighted Cox proportional hazards regression analysis and exposure-response curves were applied.

**Results:**

This included 5,412 adults, representing 76,479,702 individuals. During a mean of 5.85 years of follow-up (31,653 person-years), 96 CVD and 356 all-cause mortality events occurred. Age and sex-adjusted survival curves showed that individuals with higher levels of serum Cu experienced increased CVD and all-cause death rates (tertiles, *p* < 0.05). Compared with the participant in tertile 1 of serum Cu (< 16.31 mol/L), those in tertile 3 (≥ 19.84 mol/L) were significantly associated with CVD mortality (HR: 7.06, 95%CI: 1.85,26.96), and all-cause mortality (HR: 2.84, 95% CI: 1.66,4.87). The dose-response curve indicated a linear relationship between serum Cu and CVD mortality (*p* -nonlinear = 0.48) and all-cause (*p* -nonlinear = 0.62). A meta-analysis included additional three prospective cohorts with 13,189 patients confirmed the association between higher serum Cu and CVD (HR: 2.08, 95% CI: 1.63,2.65) and all-cause mortality (HR: 1.89, 95%CI: 1.58,2.25).

**Conclusion:**

The present study suggests excessive serum Cu concentrations are associated with the risk of CVD and all-cause mortality in American adults. Our findings and the causal relationships require further investigation.

**Graphical Abstract:**

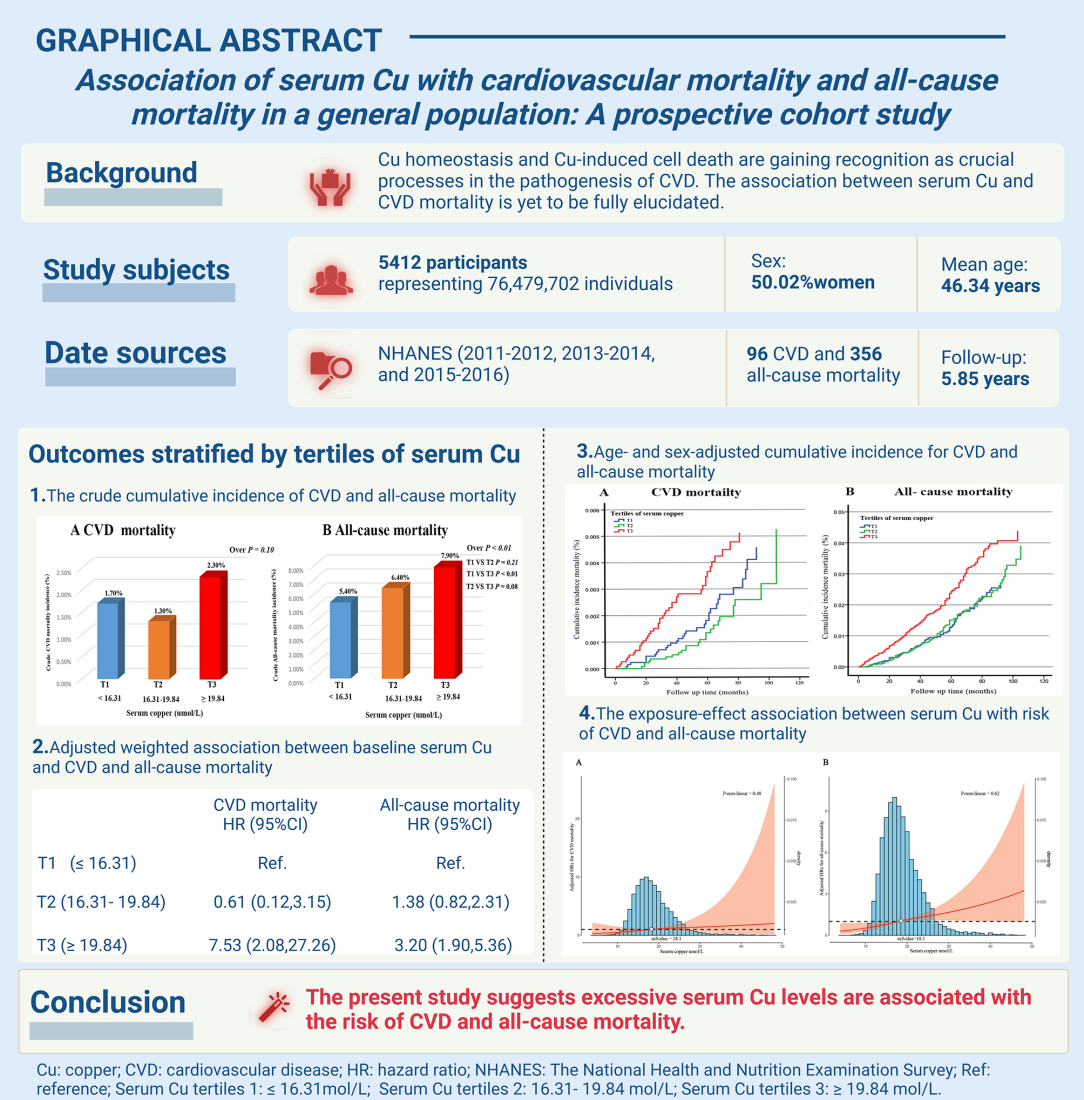

**Supplementary Information:**

The online version contains supplementary material available at 10.1186/s12889-023-17018-3.

## Introduction

The World Health Organization reported that cardiovascular disease (CVD) is currently a major contributor to global morbidity and mortality, accounting for an estimated 31% of all deaths worldwide [1]. Furthermore, CVD also causes great financial burden, straining healthcare spending [2]. Tremendous efforts are being made to prevent CVD, including identifying conventionally hazardous factors and changing unfit lifestyles. Thus, it is important to confirm new changeable hazardous factors for the prevention of CVD. Emerging evidence suggests that long-term exposure to metal elements (arsenic, cadmium, titanium, lead, etc.) could have advantageous or adverse impacts on human physical conditions. For example, there were reports of an association between plasma cadmium concentration and the risks of total and specific-cause deaths [3], and the blood titanium and arsenic concentrations and CVD incidence [4].

Copper (Cu), as an auxiliary factor of several enzyme activities [5], and angiogenesis [6], is an important metal for maintaining balance and biological function in the body [7], comparable to zinc and iron, and has a crucial role in the metabolic processes of humans. Cu deficiency in myocardial cells can reduce Cu chaperone levels and inhibit the activity of cytochrome c oxidase, impairing the systolic function of the left ventricle in diabetic cardiomyopathy [8]. Meanwhile, patients with diabetic cardiomyopathy have higher circulating Cu concentrations and lower intracellular myocardial Cu + levels [9]. However, the levels of Cu intracellularly increased, and redundant Cu ions combined with mitochondrial proteins caused proteotoxic stress-mediated cell death [10]. A meta-analysis, including 13 studies and 1,504 individuals, found that the serum Cu levels in heart failure (HF) patients were higher compared to control subjects (standard mean difference = 0.982) [11].

As we previously reviewed, the imbalance of Cu content in the human body has been associated with CVD, such as coronary heart disease (CHD), stroke, hypertension, ischemia-reperfusion injury, and HF [12, 13]. A nested case-control study that enrolled 2,410 patients with hypertension showed a positive association between plasma Cu levels and the risk of total stroke, with an odds ratio of 1.49 in the highest versus the lowest quartile of plasma Cu. [14]. A second analysis from the Kuopio Ischemic Heart Disease prospective cohort involving 2,492 adult males without venous thromboembolism showed participants with plasma Cu concentrations (≥ 1.18 mg/L) had higher CHD risk than those with lower plasma Cu concentrations (< 1.02 mg/L) [15]. However, the evidence on the link between circulating Cu and CVD or all-cause mortality is limited. We hypothesize that there is a significant association between excessive serum Cu levels and CVD and all-cause mortality. Thus, by using the dataset from the National Health and Nutrition Examination Survey (NHANES), we aim to examine the association between serum Cu and CVD mortality and all-cause mortality in American adults.

## Materials and methods

### Data source and study subjects

NHANES is a continuous, nationally representative survey aimed at evaluating the health and nutritional status of the non-institutionalized civilian population in the U.S. [16]. The survey is performed each 2 years by the National Center for Health Statistics (NCHS) of the Centers for Disease Control and Prevention (CDC). Only the 2011–2012, 2013–2014, and 2015–2016 NHANES cycles measured the participants’ serum Cu concentrations. We therefore only selected data from those three cycles. The data from these three cycles were linked to public-use mortality data. This provided death follow-up information from the survey participants’ data until December 31, 2019.

**Fig. 1:**
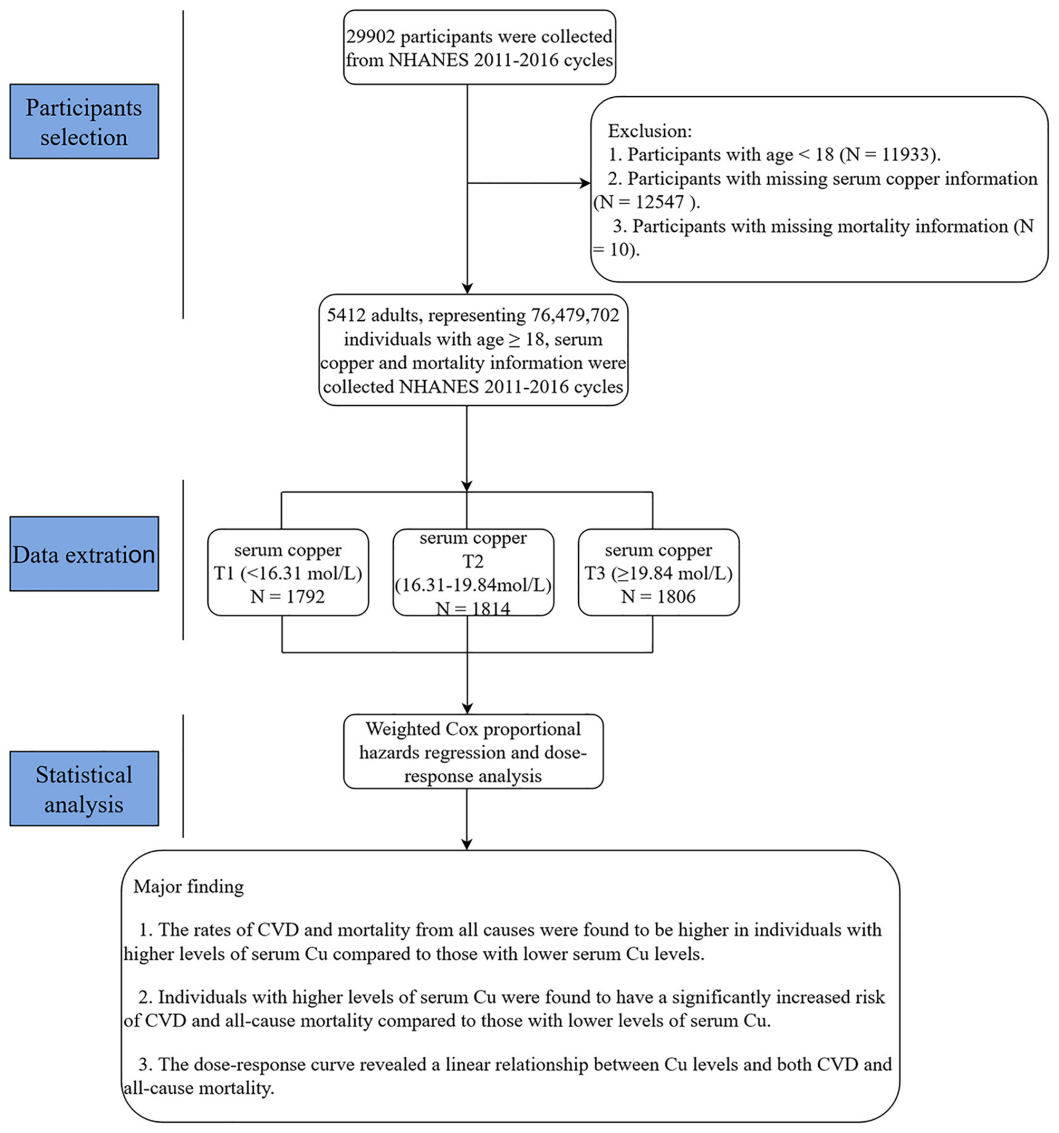
Study selection from the NHANES 2011–2016; workflow and major findings of this study Abbreviations: NHANES: National Health and Nutrition Examination Survey; CVD: cardiovascular disease; Cu: copper.

The selection process of participants in this prospective cohort study is shown in Fig. 1. Altogether 29,902 subjects were initially included in NHANES (2011–2016) datasets. Participants with ages < 18 years (n = 11,933), missing data on serum Cu (n = 12,547), or mortality (n = 10) were excluded. Finally, 5,412 represented 76,479,702 participants were included in this study.

### Measurements

During the household interview, the questionnaires were applied to evaluate the baseline demographic data, including age, sex, race/ethnicity, educational status, smoking, the ratio of family income to poverty (low: < 1.30, moderate: 1.31 to 3.50; high: ≥ 3.5), and medical history.

Trained staff collected weight and height data at the Mobile Examination Center. Body mass index (BMI) was calculated as weight (kg) divided by the square of height (m). Participants’ systolic blood pressure (SBP) and diastolic blood pressure (DBP) were carefully measured, and the final value was the average of three consecutive BP measurements. Smoking status was determined by responses about whether subjects reported having smoked more than 100 cigarettes in their lifetime and if they did so now. Drinking status was determined by responses about having more than 12 drinks over their lifetime and having more than 12 drinks in the previous year. Physical activity (PA) was ascertained by the responses whether a participant was vigorous or moderate in recreational activities.

Furthermore, total cholesterol (TC), high-density lipoprotein cholesterol (HDL-C), fasting triglycerides (TG), low-density lipoprotein cholesterol (LDL-C), fasting glucose, albumin, hemoglobin A1c (HbA1c), uric acid (UA), and creatinine, were measured at baseline when the participants provided their blood samples at the mobile examination center. The Chronic Kidney Disease Epidemiology Collaboration equation was applied to count the estimated glomerular filtration rate (eGFR) [17].

HF, CHD, and stroke were defined based on self-reported history. Hypertension was defined as a self-reported history of hypertension or treatment with anti-hypertension medication and/or SBP more than 130 mm Hg and/or DBP more than 80 mm Hg [18]. Diabetes mellitus was defined as having a fasting serum glucose of ≥ 126 mg/dL (≥ 7.0 mmol/L) [19], having an HbA1c level greater than 6.5% [20], having been diagnosed with diabetes, or being on anti-diabetic medications (insulin or oral hypoglycemic agents). Dyslipidemia was considered as self-reported having a dyslipidemia history or treatment with cholesterol-lowering medications and had a TC ≥ 6.2 µmol/L and LDL-C ≥ 4.1 µmol/L [21].

### Measurement of serum Cu

The baseline serum Cu levels of participants were measured by inductively coupled plasma dynamic reaction cell mass spectrometry (ICP-DRC-MS) [22]. The detection lower limit (LLOD) for Cu was 0.39 µmol/L, and all recorded values met or exceeded this threshold, representing 100% of the proportions.

### Assessment of mortality outcomes

The mortality of each participant in NHANES was determined through a probabilistic record match to death certificate records from the National Death Index (https://www.cdc.gov/nchs/data-linkage/). Vital status was ascertained from additional sources, including information obtained from linkages with the U.S. Social Security Administration and/or by active follow-up of survey participants. Follow-up time was counted from the baseline examination date (2011–2016) until the registered date of death or the end of the study (December 31, 2019). The primary outcomes of interest in this study were mortality from CVD and all causes (codes I00-I09, I11, I13, and I20-I51), and compliance with the codes of ICD-10 (International Statistical Classification of Diseases, 10th revision).

### Covariates

The selected potential covariates included age, sex, marital status (unmarried, married, other), education (primary school graduate or below, middle/high/special school, college graduate or above), BMI, eGFR, HbA1c, UA, moderate PA, drinking status, smoking status, and history of the comorbidities (CHD, diabetes mellitus, hypertension, dyslipidemia).

### Statistical analysis

Continuous variables were displayed as weighted means and standard error (SE), and categorical variables were described as weighted frequency percentages. The population was divided into three groups based on baseline serum Cu tertiles: tertile 1 (< 16.31 mol/L), tertile 2 (16.31–19.84 mol/L), and tertile 3 (≥ 19.84 mol/L). Preliminary descriptive analyses of differences in characteristics among the three groups were performed using the chi-square test for categorical variables and the Kruskal–Wallis H test for continuous variables.

Age and sex-adjusted CVD and all-cause cumulative incidence curves by tertiles of serum Cu were plotted. The weighted Cox proportional hazards regression models were applied to evaluate the association between serum Cu (tertiles) and the risk of CVD and all-cause mortality. A continuous relationship was assessed by Z-score serum Cu. The directed acyclic graph was used to select covariates based on knowledge as potential confounders, and included sex, age, marital status, HbA1c, UA, eGFR, education, CHD, BMI, hypertension, diabetes, hyperlipidemia, smoking status, drinking status, and moderate PA (Figure S1). The results were expressed as hazard ratios (HRs) and the corresponding 95% confidence intervals (CIs) with three pre-established models. Model I was adjusted for age, sex, marital, education, BMI, eGFR, HbA1c, and UA; Model II was adjusted for Model I plus CHD, diabetes, hypertension, dyslipidemia, moderate PA, smoking status, and drinking status. Restricted cubic spline curves were adopted to appraise their dose-response relationship [23].

The stratified analyses were conducted in the following pre-defined variables, including sex, age (< 65 vs. ≥ 65 years old), BMI (< 30 vs. ≥ 30 kg/m2), eGFR (< 90 vs. ≥ 90 mL/min/1.73 m2), hypertension (yes vs. no), diabetes (yes vs. no), dyslipidemia (yes vs. no), drinking status (never vs. former vs. current), and moderate PA (yes vs. no). In the subgroup analysis, we used the highest tertile of serum Cu compared to the lowest tertile.

A competing risk analysis was conducted to analyze serum Cu associated with CVD death. Non-CVD death was regarded as a competing event [24]. We conducted a propensity-score matching (PSM). First, the propensity score was calculated by creating a propensity score model, which is a multivariable COX regression analysis. The variables for this model were derived from baseline characteristics with p-values < 0.2 in Table S1. Second, participants in tertile 3 of serum Cu were matched 1:1 by log propensity score to those in tertiles 1–2, using the nearest neighbor matching algorithm, with a caliper difference of 0.2 [25].

A multiple-imputation analysis by the Markov-chain Monte Carlo method was used to account for missing data (BMI, education, eGFR, drinking status, hypertension, and age) [24].

We further performed a meta-analysis of prospective studies to systematically evaluate serum Cu associated with CVD death and all-cause death in the general population. Three databases (PubMed, Embase, and the Cochrane Library) up until June 2022 were sought out eligible studies by using the following MeSH words: (“Trace Elements” OR “copper”) AND (“death”). The Q statistics and Tau2 statistics were applied to estimate heterogeneity across studies. The included study’s quality was evaluated using Newcastle-Ottawa evaluation scale (NOS) scores; scores of ≥ 7 indicated acceptable quality [26].

All analyses accounted for the complexity of the sample design by using a survey package in R software to determine primary sampling units, strata, and weights. All samples were estimated using Cu, selenium and zinc weights, and variance was estimated using masked variance units [27]. A two-tailed P < 0.05 was considered statistically significant.

## Results

### Baseline characteristics by tertiles of serum Cu

As illustrated in Table 1 the mean (SE) age of the subjects was 46.3 years (0.44). The female proportion was 50.02% (2,707). The mean (SE) value was 18.54 µmol/L (0.19) for serum Cu, 28.99 kg/m^2^ (0.21) for BMI. Most participants were non-Hispanic whites (65.54%). The subjects reported as follows: 19.06% currently smoking, 77.73% currently drinking, 54.88% married, 63.38% college graduate or above, 23.36% g poor economic status, 43.57% participation in moderate PA, and 26.03% participation in vigorous PA. History of hypertension, diabetes, dyslipidemia, HF, CHD, stroke was 44.94%, 14.41%, 41.51%, 2.37%, 3.45%, and 2.48%, respectively.

**Table 1:** **Weighted Baseline Characteristics of the association of serum copper with cardiovascular mortality and all-cause mortality, Nation Health and Nutrition Examination Survey 2011–2016**

Characteristic	Population estimates	Observations
**Overall**	76,479,702	5412
Copper, µmol/L	18.54 (0.19)	5412
Age, year	46.34 (0.44)	5412
Female	50.02%	2707
BMI, kg/m^2^	28.99 (0.21)	5344
Waist circumference, cm	99.44 (0.55)	5131
DBP, mm Hg	69 (0.44)	4867
SBP, mm Hg	123 (0.61)	4867
**Smoke status**		
Never smoke	56.53%	3059
Former smoke	24.41%	1321
Current smoking	19.06%	1031
**Drinking status**		
Never drink	12.30%	666
Former drink	9.98%	504
Current drinking	77.73%	4206
**Race**		
Mexican American	8.73%	472
Other Hispanic	6.65%	360
Non-Hispanic White	65.54%	3547
Non-Hispanic Black	10.94%	592
Other Race	8.14%	440
**Marital status, % (n)**		
Never married	19.68%	1065
Married	54.88%	2970
Other	25.44%	1377
**Education status, % (n)**		
Primary school graduate or below	6.04%	327
Middle/high/special school	30.57%	1654
College graduate or above	63.38%	3430
**The ratio of family income to poverty, % (n)**		
Low	23.36%	1264
Moderate	38.35%	2074
High	38.59%	2088
**Physical activity**		
Moderate, % (n)	43.57%	2358
Vigorous, % (n)	26.03%	1409
**Laboratory results**		
TC, mmol/L	4.94 (0.03)	5357
TG, mg/dL	1.34 (0.03)	2472
HDL-C, mmol/L	1.41 (0.01)	5357
LDL-C, mg/dL	2.93 (0.03)	2435
Fasting glucose, mg/dL	5.88 (0.04)	2560
Cr, umol/L	76.28 (0.62)	5344
eGFR, ml/min/1.73m2	122.64 (1.24)	5284
UA, umol/L	323.62 (2.29)	5343
HbA1c, %	5.63 (0.03)	5406
Selenium, umol/L	1.66 (0.01)	5411
Zinc, umol/L	13.53 (0.08)	5411
**Disease**		
Hypertension	44.94%	2432
Diabetes	14.41%	780
Dyslipidemia	41.51%	2246
HF	2.37%	128
CHD	3.45%	187
Stroke	2.48%	134

Note: Data are expressed as mean (SE) and numbers (percentage) as appropriate. All estimates were weighted to be nationally representative

Abbreviations: SE: standard error; BMI: body mass index; DBP: diastolic blood pressure; SBP: systolic blood pressure; HbA1c: glycated hemoglobin; TG: triglycerides; TC: total cholesterol; LDL-C: lower-density lipoprotein cholesterol; HDL-C: high-density lipoprotein cholesterol; Cr: creatinine; UA: uric acid; eGFR: estimated glomerular filtration rate. HF: heart failure; CHD: coronary heart disease

Table S1 displays the baseline characteristics of the cohort of subjects by baseline serum Cu tertiles. Participants with higher serum Cu levels were older, female, non-Hispanic black, had a higher BMI and waist circumference, were less likely to be married, had a lower education level, more likely to be a current smoker, less likely to be a current drinker, exercised less, had higher TC, LDL-C, fasting glucose, eGFR, HbA1c, lower UA (*p* < 0.05), and had a higher prevalence of hypertension, diabetes, dyslipidemia, HF, CVD, and stroke but this was not significant (*p* > 0.10). The serum Cu levels of females were higher than those of males.

### CVD and all-cause survival rate across baseline serum Cu level

Figure S2 demonstrates the crude cumulative incidence of CVD and all-cause mortality based on tertiles of baseline serum Cu. The CVD mortality in the three groups is similar (p = 0.10). However, when comparing the results to the first tertile (≤ 16.31 µmol/L), all-cause mortality in the second tertile (16.31–19.84 µmol/L) is significant higher (p < 0.01). Furthermore, while the all-cause mortality in the third tertile (≥ 19.84 µmol/L) demonstrates an increasing trend compared to the second tertile, it does not reach statistical significance (*p* = 0.08).

**Fig. 2:**
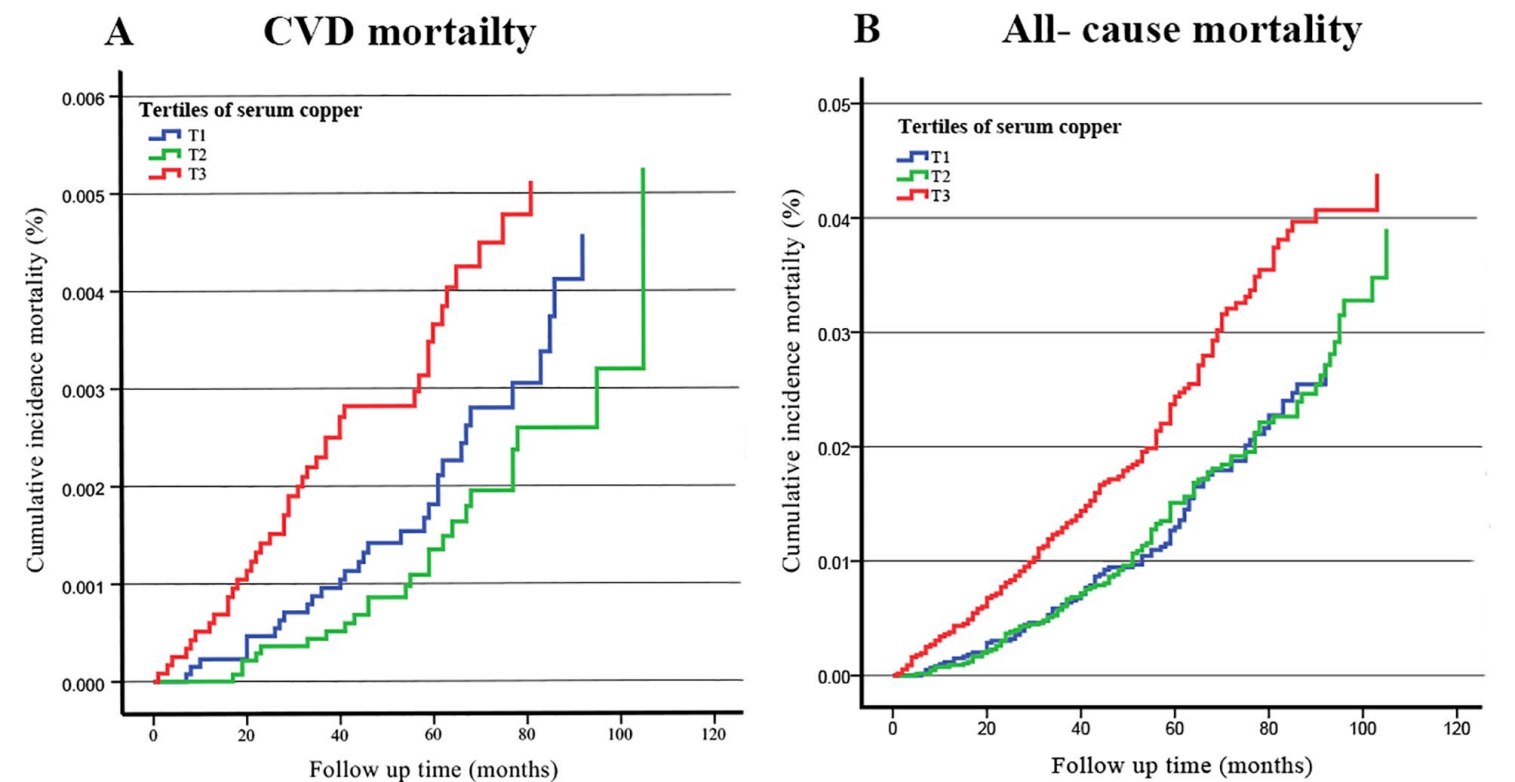
Age- and sex-adjusted Kaplan–Meier curves of tertiles of serum Cu with CVD and all-cause mortality. (**A**) CVD mortality. (**B**) all-cause mortality Abbreviations: Cu: copper; CVD: cardiovascular disease

Figure 2 illustrates age- and sex-adjusted cumulative incidence for CVD and all-cause death based on tertile of baseline serum Cu. Participants with higher serum Cu levels experienced increased CVD and all-cause mortality event rates (*p* < 0.05). Compared with the subjects in tertile 1, those in tertile 3 of serum Cu levels have increased all-cause mortality event rates (*p* < 0.01) but have similar CVD mortality event rates (*p* > 0.05), while tertile 2 have similar CVD and all-cause mortality event rates (*p* > 0.05). Tertile 3 had higher CVD and all-cause mortality event rates than tertile 2 (*p* < 0.01).

#### Associations of serum Cu with mortality from CVD and all-cause death

Table 2 demonstrates the crude and adjusted association between baseline serum Cu and CVD and all-cause mortality. In the crude model, compared to the participants in tertile 1, those in tertile 3 were markedly associated with CVD mortality (HR: 3.29, 95%CI: 1.29,8.40), but not for tertile 2 (HR: 0.56, 95%CI: 0.13,2.41). In the fully adjusted model, those in tertile 3 were significantly associated with risk of CVD mortality (HR: 7.06, 95%CI: 1.85,26.96), but not for tertile 2 (HR: 0.60, 95%CI: 0.11,3.30).

**Table 2:** The associations of serum copper with cardiovascular mortality and all-cause mortality, Nation Health and Nutrition Examination Survey 2011–2016

Copper, µmol/L	Cases/sample size	Crude Model HR (95%CI)	P	Model I HR (95%CI)	P	Model II HR (95%CI)	P
**CVD mortality**							
Per 1 SD increase	96/5412	1.40 (1.16,1.70)	< 0.01	2.58 (1.56,4.26)	< 0.01	2.69 (1.70,4.28)	< 0.01
Tertiles							
T1 (≤ 16.31)	31/1792	Ref.		Ref.		Ref.	
T2 (16.31–19.84)	24/1814	0.56 (0.13,2.41)	0.44	0.59 (0.13,2.56)	0.48	0.60 (0.11,3.30)	0.55
T3 (≥ 19.84)	41/1806	3.29 (1.29,8.40)	0.01	5.02 (1.60,15.71)	< 0.01	7.06 (1.85,26.96)	< 0.01
P for trend		0.01		< 0.01		< 0.01	
**All-cause mortality**							
Per 1 SD increase	356/5412	1.28 (1.14,1.43)	< 0.01	1.91 (1.57,2.33)	< 0.01	1.76 (1.40,2.21)	< 0.01
Tertiles							
T1 (≤ 16.31)	97/1792	Ref.		Ref.		Ref.	
T2 (16.31–19.84)	116/1814	1.47 (0.68,2.30)	0.09	1.42 (0.88,2.27)	0.15	1.17 (0.70,1.97)	0.55
T3 (≥ 19.84)	143/1806	2.32 (1.59,3.38)	< 0.01	3.11 (1.83,5.29)	< 0.01	2.84 (1.66,4.87)	< 0.01
P for trend		< 0.01		< 0.01		0.02	

Note: Crude Model: unadjusted any factor

Model I: multi-factor model adjusted for age, gender, marital, education, BMI, eGFR, HbA1c, and UA.

Model II: multi-factor model adjusted for Model I+CHD, diabetes mellitus, hypertension, dyslipidemia, moderate PA, smoking status, and drinking status

Abbreviations: 95% CI: 95% confidence interval; HR: hazard ratio; Ref: reference; BMI: body mass index; UA: uric acid; PA: Physical activity; CHD: coronary heart disease; HbA1c: glycated hemoglobin; CVD mortality: cardiovascular disease mortality; eGFR: estimated glomerular filtration rate

For all-cause mortality, tertile 3 was markedly associated with risk of all-cause mortality (HR: 2.32, 95% CI: 1.59,3.38), but not a significant association for those with tertile 2 (HR: 1.47, 95% CI: 0.68, 2.30). After full adjustments, the results did not significantly change (tertile 3 h: 2.84, 95%CI: 1.66,4.87; tertile 2: HR: 1.17, 95%CI: 0.70,1.97).

The continuous variables analysis (per 1 SD increase) demonstrated a positive association for CVD mortality (HR: 2.69, 95% CI: 1.70,4.28) and all-cause mortality (HR: 1.76, 95% CI: 1.40,2.21) after full adjustments, respectively.

### Curve-fitting association of serum Cu with mortality

**Fig. 3:**
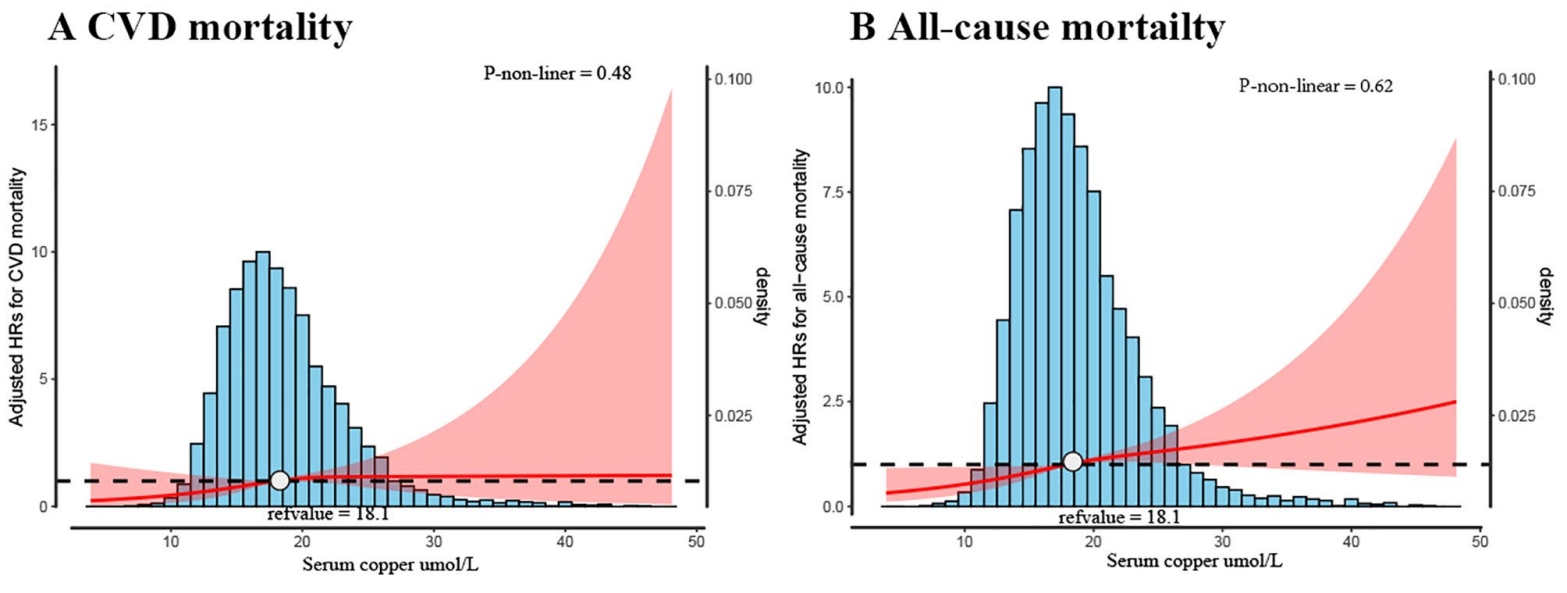
Cox proportional hazards regression model of serum copper with CVD (**A**) and all-cause mortality (**B**). The serum copper distribution was depicted using the histogram. Adjusted hazard ratios (95% CI) for CVD and all-cause mortality are shown with red curves. The serum copper level considered for this analysis was 18.1 µmol/L. The model cubic spline curves have 3 knots. Adjustments included age, sex, marital, education, BMI, eGFR, HbA1c, UA, CHD, diabetes mellitus, hypertension, dyslipidemia, moderate PA, smoking status, and drinking status Abbreviations: CVD: cardiovascular disease; BMI: body mass index; eGFR: estimated glomerular filtration rate; HDL-C: high-density lipoprotein cholesterol; UA: uric acid; CHD: coronary heart disease; PA: Physical activity

The exposure-effect association between serum Cu and the risk of CVD and all-cause mortality is presented in Fig. 3. There was a nearly linear association between serum Cu level CVD mortality (*p*-nonlinear = 0.48) and all-cause mortality (*p*-nonlinear = 0.62) (Fig. 3A-B), with the risk of CVD and all-cause mortality doubled when serum Cu levels was approximately 18.1 µmol/L.

### Subgroup analyses and sensitivity analyses

**Fig. 4:**
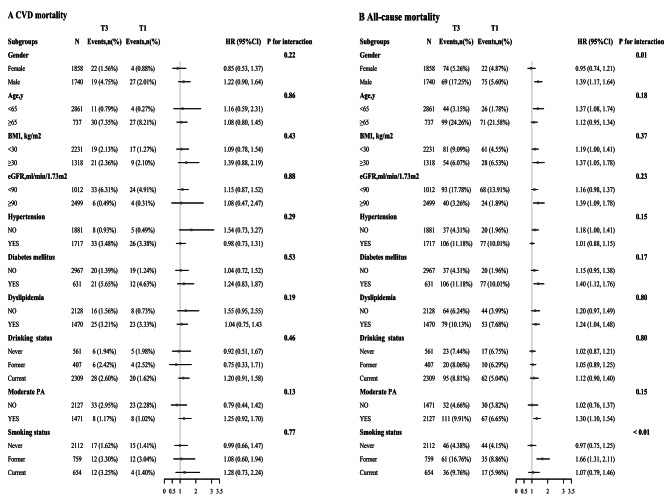
Association of serum copper (T3 vs. T1) with CVD and all-cause mortality in different subgroups. A: CVD mortality; B: all-cause mortality. The results are adjusted for age, sex, marital, education, BMI, eGFR, HbA1c, UA, CHD, diabetes mellitus, hypertension, dyslipidemia, moderate PA, smoking status, and drinking status, if the above variables are not adjusted Abbreviations: CVD: cardiovascular disease; BMI: body mass index; eGFR: estimated glomerular filtration rate; HDL-C: high-density lipoprotein cholesterol; UA: uric acid; PA: Physical activity

Subgroup analyses were employed based on sex, age, BMI, eGFR, hypertension, dyslipidemia, diabetes mellitus, smoking status, drinking status, and moderate PA (Fig. 4). For CVD mortality, the stratified analyses did not find any significant interactions between interested variables and serum Cu levels (all *p* interactions > 0.1). (Fig. 4A). The association between serum Cu and all-cause mortality was significantly stronger in males (HR: 1.44, 95% CI: 1.22,1.70) than in females (HR: 0.99, 95% CI: 0.78–1.26; *p* interaction = 0.01). The association between serum Cu and all-cause mortality was higher in former smokers (HR: 1.66, 95% CI: 1.31–2.11) than in those who had never smoked (HR: 0.97, 95% CI: 0.75–1.25) and those who were current smokers (HR: 1.07; 95% CI: 0.79–1.46; p interaction < 0.01). None of the resting variables significantly modified their association (all *p* interactions > 0.10). (Fig. 4B)

We performed multiple sensitivity analyses to evaluate the robustness of our results. Firstly, to account for zinc and selenium. We further adjusted for serum zinc and selenium to verify the association between serum Cu and CVD and all-cause mortality. As presented in Table S2 and Table S3, the Cox regression analyses showed confirmatory results. Secondly, competing for-risk model again confirmed the positive association between Cu level and CVD mortality (all *p* < 0.05, Table S4). Third, sensitivity analyses of propensity score matching that balanced the baseline characteristics by serum Cu further remained confirmatory (Tables S5-S6). Finally, results from multiple imputation for missing data were consistent with the main results (Table S7).

#### Meta-analysis

**Fig. 5:**
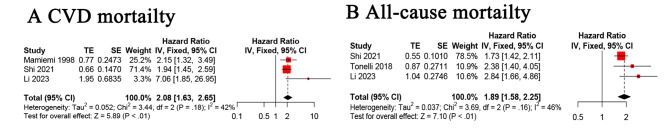
Forest plot of the pooled results between serum Cu and CVD and all-cause mortality by Fixed-effects model (highest vs. lowest). A: CVD mortality; B: All-cause mortality Note: Li 2023 refer to present study. TE: logHR; SE: selogHR. Cu: copper; CVD: Cardiovascular diseases. Referents for lowest of serum Cu were the individuals reporting with lowest of serum Cu within the specific study

We included additional 3 prospective cohorts [28–30] with acceptable study quality involving 13,189 individuals. The baseline characteristics and quality of included cohorts is shown in Tables S8-S9. As shown in Fig. 5, the summary results showed significant associations between serum Cu and CVD mortality (HR: 2.08, 95% CI: 1.63,2.65, I^2^ = 42%, *p* for Q test *= 0.18*) and all-cause mortality (HR: 1.89, 95%CI: 1.58,2.25, I^2^ = 46%, *p* for Q test *= 0.16*) (highest vs. lowest).

## Discussion

### Major findings

This nationally representative prospective cohort showed that individuals with higher levels of serum Cu have higher CVD and all-cause death than those with lower levels of serum Cu. We also discovered that elevated serum Cu was associated with CVD mortality (HR: 7.53) and all-cause mortality (HR: 3.20). Furthermore, a positive linear exposure-effect relationship further strengthens their association, and this finding is robust in stratified and sensitivity analyses. In addition to our findings, we conducted a meta-analysis that further supported the association between Cu and CVD mortality (HR: 2.08), as well as all-cause mortality (HR: 1.89). These results were consistent with our findings, thus enhancing the reliability and validity of our study.

### Comparisons with previous studies

Several previous studies described associations between serum Cu and CVD and all-cause death, but their populations were not nationally representative. A cohort study that included 3,252 participants [31] demonstrated that the highest blood Cu level was positively associated with a raised risk of CVD (HR: 1.49; 95% CI: 1.10–2.01) and all-cause mortality (HR: 1.30; 95% CI: 1.03–1.66). A cohort study from France [32] of 4035 men aged 30 to 60 years at baseline found that the highest plasma Cu level was clearly associated with all-cause death (RR: 1.5; 95%CI: 1.1–2.1) but not with CVD death (RR: 1.3; 95%CI: 0.6–2.8) after 17.8 years of follow-up. There are some reasons that may help explain this. First, the participants in this study are all men. Second, some research [33] demonstrated that the association between plasma Cu and cancer or CHD death might be sex-dependent. The population sample selected in our study is more nationally representative, with fewer immigrants and larger numbers of people included. The results obtained are positive, in line with most previous studies. For example, in a prospective study including 58,646 healthy Japanese aged 40 to 79 years, Eshak et al. demonstrated dietary Cu intake to be positively associated with CVD mortality [34]. Additionally, Marniemi et al. [28] indicated that that the highest serum Cu levels were associated with a raised risk of CVD mortality (RR: 2.15, 95% CI: 1.32–3.48) after 13 years of follow-up in 480 community seniors over age 65 years.

We further demonstrated that the highest serum Cu level was associated with CVD and all-cause mortality in multivariate Cox regression. Previous studies have shown that trace elements such as serum zinc, magnesium, and selenium may all be important cofounders for cardiovascular and all-cause mortality [35–37]. We further adjusted serum zinc and selenium for sensitivity analysis, and the results remined confirmed, which makes our conclusion more robust. Meanwhile, the linear exposure-response relationship observed in our study aligns with prior research from Finland, which also found a linear exposure-effect relationship between CVD death risk and blood Cu concentration [38].

### Underlying mechanism

The potential mechanisms that may explain our findings is poorly understood, and several hypotheses have been proposed. First, Cu is involved in oxidative damage. Cu is bound to superoxide dismutase 1 (SOD1) to detoxify reactive oxygen species (ROS) and sustain Cu homeostasis [39]. Some researchers have found that the level of SOD1 causes oxidative stress [40, 41]. In addition, the copper chaperone for superoxide dismutase (CCS) was used to deliver Cu to SOD1, and the level of CCS was based on cellular Cu status, and when Cu levels are elevated, the biodegradation of CCS increases [42]. Secondly, Cu may also be involved in inflammatory processes [43]. A cross-sectional study found that an increase in the copper/zinc ratio possibly exacerbates inflammation [44]. The higher levels of redox metals, such as Cu, can promote ROS formation and play a role as mediators of inflammation [45]. An enhanced response to oxidative stress, a chronic inflammatory state, and Cu overburden has been observed in pathological conditions in both animal models and humans [46].

Researchers have recently reported a novel form of cell death-Cu-dependent, distinguishable from familiar death mechanisms, and reliant upon mitochondrial respiration. Cu-dependent death happens through straight engagement of Cu with lipoylated constituents of the tricarboxylic acid cycle, lead to lipoylated protein accumulation and consequent iron-sulfur cluster protein l depletion, which causes proteotoxic stress and finally cell death [10]. Since blood trace element levels could inaccurately reflect dietary intake, the intricate metabolic and pathological mechanisms associated with blood trace element levels or diet are complex and reflect, for example, an individual’s genetic susceptibility. Therefore, there may not be a single association between micronutrient serum status or diet and CVD mortality.

### Clinical practice

Based on a large general population cohort study, we examined the association between serum Cu and the risk of CVD and all-cause death in adult Americans. Cu is an important micronutrient in the human body and its fluctuation in the body may have an important effect. Our study demonstrates that higher serum Cu levels increase CVD mortality and all-cause death independently of other cardiovascular risk factors. As the mechanism of Cu toxicity in humans has not yet been established, additional research is therefore necessary to investigate this question.

### Strengths and limitations

This study has some unique strengths. First, it was a nationally representative prospective cohort, with a large sample size. Thus, the well-designed cohort allowed us to adjust for a multitude of potential confounding factors. Subgroup analyses, sensitivity analyses and meta-analysis further showed the robustness of our results. However, certain limitations of this study should be acknowledged. First, our study is observational and cannot indicate causality. Secondly, the determination of specific causes of mortality in the NHANES relies on death certificates without confirmation through autopsy, potentially leading to some misclassification. Third, serum Cu fluctuated over time, and serum Cu measurement only at baseline without subsequent measurement may not accurately reflect long-term exposure. Fourth, the interaction between Cu and other metals in influencing CVD requires further investigation. Finally, the study population was aged over 18 and the results may be different in children.

## Conclusion

In the context of CVD, Cu homeostasis and Cu-induced cell death are areas of increasing interest and research. This nationally representative longitudinal study reveals significant associations between serum Cu levels and the risk of CVD and all-cause mortality, independent of established risk factors. Those with excessive serum Cu should possibly be given more attention for CVD prevention. Our findings suggest the need for further investigation into the potential causal relationship between excessive serum Cu and the risk of CVD events. Clinical and experimental studies are required to delve deeper into this association.

### Electronic supplementary material

Below is the link to the electronic supplementary material.

Supplementary Material 1

## Data Availability

The datasets generated and/or analyzed during the current study are available in the NHANES repository, https://www.cdc.gov/nchs/nhanes/.

## Abbreviations

Cu: Copper
CVD: Cardiovascular disease
NHANES: National Health and Nutrition Examination Survey
HRs: Hazard ratios
95% CI: 95% Confidence Intervals
NCHS: National Center for Health Statistics
CDC: Centers for Disease Control and Prevention
DMC: Diabetic cardiomyopathy
CHD: Coronary heart disease
BMI: Body mass index
BP: Blood pressure
PA: Physical activity
TC: Total cholesterol
HDL-C: High-density lipoprotein cholesterol
TG: Triglycerides
LDL-C: Low-density lipoprotein cholesterol
HbA1c: Hemoglobin A1c
UA: Uric acid
CKD-EPI: Chronic Kidney Disease Epidemiology Collaboration
eGFR: Estimated glomerular filtration rate
ICP-DRC-MS: Inductively coupled plasma dynamic reaction cell mass spectrometry
HF: Heart failure
CHD: Coronary heart disease
SE: Standard error
RR: Relative risk
NOS: Newcastle-Ottawa evaluation scale
Zn: Zinc
CCS: Copper chaperone for superoxide dismutase
SOD1: Superoxide dismutase1
ROS: Reactive oxygen species
