# Supplementary material for: Association of serum copper (Cu) with cardiovascular mortality and all-cause mortality in a general population: a prospective cohort study
Source: BMC Public Health. 2023 Nov 1;23:2138. doi: 10.1186/s12889-023-17018-3 (PMC10621106; doi:10.1186/s12889-023-17018-3)
Supplement: Supplementary file 1 — Supplementary Material 1 [file 12889_2023_17018_MOESM1_ESM.docx]

**Supplement material**

**Table S1 Baseline characteristics by the Tertile of the Copper of adult Americans in the cohort from the Nation Health and Nutrition Examination Survey 2011-2016**

**Table S2 The associations of serum copper with cardiovascular and all-cause mortality after adjusted serum zinc in the cohort from the Nation Health and Nutrition Examination Survey 2011-2016**

**Table S3 The associations of serum copper with cardiovascular and all-cause mortality after adjusted serum selenium in the cohort from the Nation Health and Nutrition Examination Survey 2011-2016**

**Table S4 Serum Cu and cause-specific mortality in the cohort from the Nation Health and Nutrition Examination Survey 2011-2016**

**Table S5 Baseline characteristics after propensity score matching of adult Americans in the cohort from the Nation Health and Nutrition Examination Survey 2011-2016**

**Table S6 The associations of serum copper with cardiovascular and all-cause mortality in the propensity score matched from the Nation Health and Nutrition Examination Survey 2011-2016**

**Table S7 Sensitive analysis of the association between the serum Cu and CVD and all-cause mortality in adult Americans based on the multiple-imputation analysis.**

**Table S8. Main characteristics of the included studies in the meta‐analysis of serum Cu and CVD and all-cause mortality**

**Table S9. Quality assessment of included studies**

**Figure S1. Directed Acyclic Graph (DAG) of the association between serum copper and CVD and all-cause mortality.**

**Figure S2. Crude death rates according to tertiles of serum copper. (A) CVD death. (B) All-cause death.**

**Table S1** **Baseline characteristics by the Tertile of the Copper of adult Americans in** **the cohort from the Nation Health and Nutrition Examination Survey 2011-2016**

| **Characteristics** | **Total**  **N = 5412** | **Tertiles of serum Cu** |  |  | **P** |
| --- | --- | --- | --- | --- | --- |
|  |  | **T1 (≤ 16.31μmol/L)**  **N = 1792** | **T2 (16.31-19.84μmol/L)**  **N =1814** | **T3 (≥ 19.84μmol/L)**  **N = 1806** |  |
| Copper, μmol/L | 18.54 (0.19) | 14.22 (0.08) | 17.97 (0.05) | 24.21 (0.22) | < 0.01 |
| Follow-up time, months | 70.75 (1.15) | 71.29 (1.49) | 72.13 (1.65) | 68.47 (1.36) | 0.06 |
| Age, year | 46.34 (0.44) | 43.59 (1.00) | 47.69 (0.69) | 47.88 (0.76) | < 0.01 |
| Female, % (n) | 50.02 (2707) | 22.74 (407) | 47.70 (865) | 79.46 (1435) | < 0.01 |
| BMI, kg/m^2^ | 28.99 (0.21) | 27.03 (0.27) | 29.04 (0.27) | 31.20 (0.43) | < 0.01 |
| Waist circumference, cm | 99.44 (0.55) | 96.01 (0.94) | 100.02 (0.65) | 102.84 (1.03) | < 0.01 |
| DBP, mm Hg | 69 (0.44) | 70 (0.72) | 69 (0.55) | 67 (0.55) | < 0.01 |
| SBP, mm Hg | 121 (0.61) | 121 (0.09) | 121 (1.00) | 121 (1.00) | 0.50 |
| **Smoke status, % (n)** |  |  |  |  | < 0.01 |
| Never smoke | 56.53 (3059) | 60.18 (1078) | 52.24 (948) | 57.52 (1039) |  |
| Former smoke | 24.41 (1321) | 24.87 (446) | 25.04 (454) | 23.14 (418) |  |
| Current smoking | 19.06 (1031) | 14.95 (238) | 22.72 (412) | 19.34 (349) |  |
| **drinking, % (n)** |  |  |  |  | < 0.01 |
| Never drink | 12.30 (665) | 12.09 (217) | 10.17 (184) | 15.07 (272) |  |
| Former drink | 9.98 (504) | 7.27 (130) | 10.22 (185) | 12.86 (232) |  |
| Current drinking | 77.73 (4207) | 80.64 (1445) | 79.61 (1463) | 72.07 (1032) |  |
| **Race, % (n)** |  |  |  |  | < 0.01 |
| Mexican American | 8.73 (472) | 9.76 (175) | 8.42 (153) | 7.93 (143) |  |
| Other Hispanic | 6.65 (360) | 6.10 (109) | 5.66 (103) | 8.46 (153) |  |
| Non-Hispanic White | 65.54 (3547) | 66.77 (1196) | 69.66 (1264) | 59.17 (1068) |  |
| Non-Hispanic Black | 10.94 (592) | 6.87 (123) | 9.23 (167) | 17.70 (320) |  |
| Other Race | 8.14 (440) | 10.49 (188) | 7.03 (127) | 6.74 (122) |  |
| **Marital status, % (n)** |  |  |  |  | < 0.01 |
| Never married | 19.68 (1065) | 22.43 (402) | 17.35 (315) | 19.37 (350) |  |
| Married | 54.88 (2970) | 57.60 (1032) | 58.98 (1070) | 47.00 (849) |  |
| Other | 25.44 (1378) | 19.97 (358) | 23.67 (429) | 33.63 (607) |  |
| **Education status, % (n)** |  |  |  |  | < 0.01 |
| Primary school graduate or below | 6.04 (327) | 4.23 (76) | 6.51 (118) | 7.51 (136) |  |
| Middle/high/special school | 30.57 (1654) | 29.74 (533) | 30.66 (556) | 31.41 (567) |  |
| College graduate or above | 63.38 (3430) | 66.03 (1183) | 62.83 (1140) | 61.08 (1103) |  |
| **The ratio of family income to poverty, % (n)** | | | | | < 0.01 |
| low | 23.36 (1264) | 19.21 (344) | 21.86 (397) | 30.02 (542) |  |
| Moderate | 38.05 (2059) | 36.99 (663) | 39.90 (724) | 37.04 (669) |  |
| high | 38.59 (2089) | 43.80 (785) | 38.23 (694) | 32.94 (595) |  |
| **Physical activity** |  |  |  |  | < 0.01 |
| Moderate, % (n) | 43.57 (2358) | 45.87 (822) | 42.68 (774) | 41.96 (758) |  |
| Vigorous, % (n) | 26.03 (1409) | 33.91 (608) | 22.96 (417) | 20.59 (372) |  |
| **Laboratory results** |  |  |  |  |  |
| TC, mmol/L | 4.94 (0.03) | 4.77 (0.05) | 5.02 (0.04) | 5.04 (0.05) | < 0.01 |
| TG, mg/dL | 1.34 (0.03) | 1.40 (0.06) | 1.35 (0.07) | 1.27 (0.03) | 0.50 |
| HDL-C, mmol/L | 1.41 (0.01) | 1.33 (0.02) | 1.43 (0.02) | 1.48 (0.02) | < 0.01 |
| LDL-C, mg/dL | 2.93 (0.03) | 2.81 (0.05) | 2.99 (0.04) | 2.98 (0.04) | < 0.01 |
| Fasting glucose, mg/dL | 5.88 (0.04) | 5.86 (0.08) | 5.88 (0.06) | 5.91 (0.08) | 0.64 |
| Cr, umol/L | 76.28 (0.62) | 82.20 (1.09) | 75.73 (1.03) | 70.12 (1.09) | < 0.01 |
| eGFR, ml/min/1.73m2 | 122.64 (1.24) | 118.10 (1.71) | 122.75 (1.92) | 127.77 (2.67) | < 0.01 |
| UA, umol/L | 323.36 (2.29) | 339.32 (4.55) | 320.81 (3.29) | 308.01 (4.71) | < 0.01 |
| HbA1c, % | 5.63 (0.03) | 5.54 (0.04) | 5.63 (0.05) | 5.73 (0.04) | < 0.01 |
| Selenium, umol/L | 1.66 (0.01) | 1.66 (0.01) | 1.66 (0.01) | 1.65 (0.01) | 0.93 |
| Zinc, umol/L | 13.53 (0.08) | 13.59 (0.12) | 13.59 (0.10) | 13.38 (0.16) | 0.26 |
| **Disease** |  |  |  |  |  |
| Hypertension, % (n) | 44.94 (2432) | 43.48 (779) | 44.81 (812) | 46.73 (843) | 0.42 |
| Diabetes, % (n) | 14.41 (780) | 14.92 (267) | 13.14 (238) | 15.23 (275) | 0.92 |
| Dyslipidemia, % (n) | 41.51 (2246) | 39.28 (704) | 41.52 (753) | 43.67 (789) | 0.23 |
| HF, % (n) | 2.37 (128) | 1.84 (33) | 2.56 (47) | 2.72 (49) | 0.31 |
| CVD, % (n) | 3.45 (187) | 4.55 (81) | 3.01 (55) | 2.81 (51) | 0.11 |
| Stroke, % (n) | 2.48 (134) | 1.92 (34) | 2.33 (41) | 3.28 (60) | 0.16 |

Note: Data are expressed as meann (SE) and numbers (percentage) as appropriate. All estimates were weighted to be nationally representative.

Abbreviations: SE: standard error; BMI: body mass index; DBP: diastolic blood pressure; SBP: systolic blood pressure; HbA1c: glycated hemoglobin; TG: triglycerides; TC: total cholesterol; LDL-C: lower-density lipoprotein cholesterol; HDL-C: high-density lipoprotein cholesterol; ALT: Alanine Aminotransferase; Cr: creatinine; UA: uric acid; eGFR: estimated glomerular filtration rate; HF: heart failure; CHD: coronary heart disease.

**Table S2 The associations of serum copper with cardiovascular and all-cause mortality after adjusted serum zinc in** **the cohort from the Nation Health and Nutrition Examination Survey 2011-2016**

| **Copper, μmol/L** | **Number of deaths/totals** | **Model I**  **HR (95%CI)** | **P** | **Model II**  **HR (95%CI)** | **P** |
| --- | --- | --- | --- | --- | --- |
| CVD mortality |  |  |  |  |  |
| Per 1 SD increase | 96/5412 | 2.69 (1.70,4.28) | < 0.01 | 2.69 (1.71,4.24) | < 0.01 |
| Tertiles |  |  |  |  |  |
| T1 (≤ 16.31) | 31/1792 | Ref. | 1.0 | Ref. | 1.0 |
| T2 (16.31- 19.84) | 24/1814 | 0.60 (0.11,3.30) | 0.55 | 0.61 (0.11,3.34) | 0.57 |
| T3 (≥ 19.84) | 41/1806 | 7.06 (1.85,26.96) | < 0.01 | 7.26 (1.89,27.87) | < 0.01 |
| P for trend |  | < 0.01 |  | < 0.01 |  |
| All-cause mortality |  |  |  |  |  |
| Per 1 SD increase | 356/5412 | 1.76 (1.40,2.21) | < 0.01 | 1.71 (1.41,2.08) | < 0.01 |
| Tertiles |  |  |  |  |  |
| T1 (≤ 16.31) | 97/1792 | Ref. | 1.0 | Ref. | 1.0 |
| T2 (16.31-19.84) | 116/1814 | 1.17 (0.70,1.97) | 0.55 | 1.13 (0.67,1.91) | 0.64 |
| T3 (≥ 19.84) | 143/1806 | 2.84 (1.66,4.87) | < 0.01 | 2.66 (1.66,4.25) | < 0.01 |
| P for trend |  | 0.02 |  | 0.02 |  |

Note: Model I: multi‐factor model adjusted for age, gender, marital, education, BMI, eGFR, HbA1c, UA, CHD, diabetes mellitus, hypertension, dyslipidemia, moderate PA, smoking status, and drinking status.

Model II: multi‐factor model adjusted for Model I, and Zinc.

Abbreviations: 95% CI: 95% confidence interval; HR: hazard ratio; Ref: reference; BMI: body mass index; UA: uric acid; PA: Physical activity; CHD: coronary heart disease; HbA1c: glycated hemoglobin; CVD mortality: cardiovascular disease mortality; eGFR: estimated glomerular filtration rate.

**Table S3 The associations of serum copper with cardiovascular and all-cause mortality after adjusted serum selenium** **in** **the cohort from the Nation Health and Nutrition Examination Survey 2011-2016**

| **Copper, μmol/L** | **Number of death/total** | **Model I**  **HR (95%CI)** | **P** | **Model II**  **HR (95%CI)** | **P** |
| --- | --- | --- | --- | --- | --- |
| CVD mortality |  |  |  |  |  |
| Per 1 SD increase | 96/5412 | 2.69 (1.70,4.28) | < 0.01 | 2.66 (1.73,4.08) | < 0.01 |
| Tertiles |  |  |  |  |  |
| T1 (≤ 16.31) | 31/1792 | Ref. | 1.0 | Ref. | 1.0 |
| T2 (16.31- 19.84) | 24/1814 | 0.60 (0.11,3.30) | 0.55 | 0.62 (0.11,3.47) | 0.59 |
| T3 (≥ 19.84) | 41/1806 | 7.06 (1.85,26.96) | < 0.01 | 7.14 (1.85,27.61) | < 0.01 |
| P for trend |  | < 0.01 |  | < 0.01 |  |
| All-cause mortality |  |  |  |  |  |
| Per 1 SD increase | 356/5412 | 1.76 (1.40,2.21) | < 0.01 | 1.75 (1.41,2.18) | < 0.01 |
| Tertiles |  |  |  |  |  |
| T1 (≤ 16.31) | 97/1792 | Ref. | 1.0 | Ref. | 1.0 |
| T2 (16.31-19.84) | 116/1814 | 1.17 (0.70,1.97) | 0.55 | 1.16 (0.70,1.94) | 0.56 |
| T3 (≥ 19.84) | 143/1806 | 2.84 (1.66,4.87) | < 0.01 | 2.81 (1.67,4.72) | < 0.01 |
| P for trend |  | 0.02 |  | 0.02 |  |

Note: Note: Model I: multi‐factor model adjusted for age, gender, marital, education, BMI, eGFR, HbA1c, UA, CHD, diabetes mellitus, hypertension, dyslipidemia, moderate PA, smoking status, and drinking status.

Model II: multi‐factor model adjusted for Model I, and selenium.

Abbreviations: 95% CI: 95% confidence interval; HR: hazard ratio; Ref: reference; BMI: body mass index; UA: uric acid; PA: Physical activity; CHD: coronary heart disease; HbA1c: glycated hemoglobin; CVD mortality: cardiovascular disease mortality; eGFR: estimated glomerular filtration rate.

**Table S4 Serum Cu and cause-specific mortality in the cohort from the Nation Health and Nutrition Examination Survey 2011-2016**

| **Copper, μmol/L** | **Number of deaths/totals** | **Crude Model**  **HR (95%CI)** | **P** | **Model I**  **HR (95%CI)** | **P** | **Model II**  **HR (95%CI)** | **P** |
| --- | --- | --- | --- | --- | --- | --- | --- |
| CVD mortality |  |  |  |  |  |  |  |
| Per 1 SD increase | 96/5412 | 1.060 (0.925, 1.216) | 0.401 | 1.105 (0.810,1.509) | 0.528 | 1.002 (1.000, 1.004) | < 0.001 |
| Tertiles |  |  |  |  |  |  |  |
| T1 (≤ 16.31) | 31/1792 | Ref. | 1.0 | Ref. | 1.0 | Ref. | 1.0 |
| T2 (16.31-19.84) | 24/1814 | 0.758 (0.459, 1.254) | 0.208 | 0.805 (0.354, 1.828) | 0.604 | 0.961 (0.960,0.963) | < 0.001 |
| T3 (≥ 19.84) | 41/1806 | 1.445 (0.916, 2.313) | 0.113 | 1.038 (0.543, 1.892) | 0.911 | 1.095 (1.090, 1.096) | < 0.001 |
| P for trend |  | < 0.001 |  | < 0.001 |  | < 0.001 |  |

Note: Crude Model: unadjusted any factor.

Model I: multi‐factor model adjusted for age, gender, marital, education, BMI, eGFR, HbA1c, and UA.

Model II: multi‐factor model adjusted for Model I, CHD, diabetes mellitus, hypertension, dyslipidemia, moderate PA, smoking status, and drinking status.

Abbreviations: 95% CI: 95% confidence interval; HR: hazard ratio; Ref: reference; BMI: body mass index; UA: uric acid; PA: Physical activity; CHD: coronary heart disease; HbA1c: glycated hemoglobin; CVD mortality: cardiovascular disease mortality; eGFR: estimated glomerular filtration rate.

**Table S5** **Baseline characteristics after propensity score matching of adult Americans in the cohort from the Nation Health and Nutrition Examination Survey 2011-2016**

| **Characteristics** | **Total**  N = 884 | **Tertiles of serum Cu** |  | **P** |
| --- | --- | --- | --- | --- |
|  |  | **T1 (≤19.84μmol/L)**  N = 442 | **T2 (> 19.84μmol/L)**  N = 442 |  |
| Copper, μmol/L | 17.09 (0.22) | 14.38 (0.11) | 19.70 (0.27) | < 0.01 |
| Follow-up time, months | 70.86 (1.09) | 71.44 (1.48) | 70.31 (1.29) | 0.51 |
| Age, year | 46.95 (0.77) | 46.29 (1.29) | 47.58 (0.96) | 0.44 |
| Female, % (n) | 30.85 (272) | 27.67 (122) | 33.91 (150) | 0.18 |
| BMI, kg/m^2^ | 28.00 (0.21) | 27.94 (0.34) | 28.06 (0.25) | 0.77 |
| Waist circumference, cm | 98.79 (0.64) | 98.54 (1.09) | 99.04(0.69) | 0.70 |
| DBP, mm Hg | 70 (0.59) | 70 (0.81) | 70 (0.72) | 0.51 |
| SBP, mm Hg | 121 (0.86) | 121 (1.15) | 121 (0.96) | 0.76 |
| **Smoke status, % (n)** |  |  |  | 0.78 |
| Never smoke | 54.00 (478) | 53.62 (237) | 54.38 (240) |  |
| Former smoke | 27.41 (242) | 27.65 (122) | 27.18 (120) |  |
| Current smoking | 18.59 (164) | 18.73 (83) | 18.44 (82) |  |
| **drinking, % (n)** |  |  |  | 0.16 |
| Never drink | 7.22 (64) | 8.19 (36) | 6.28 (28) |  |
| Former drink | 6.22 (55) | 7.65 (34) | 4.85 (21) |  |
| Current drinking | 86.56 (765) | 84.15 (372) | 88.86 (393) |  |
| **Race, % (n)** |  |  |  | 0.06 |
| Mexican American | 8.62 (77) | 9.70 (44) | 7.58 (33) |  |
| Other Hispanic | 6.02 (53) | 5.76 (25) | 6.26 (28) |  |
| Non-Hispanic White | 69.46 (614) | 70.00 (309) | 68.95 (305) |  |
| Non-Hispanic Black | 8.30 (73) | 6.40 (28) | 10.12 (45) |  |
| Other Race | 7.60 (67) | 8.14 (36) | 7.09 (31) |  |
| **Marital status, % (n)** |  |  |  | 0.10 |
| Never married | 20.79 (184) | 20.17 (89) | 21.38 (95) |  |
| Married | 59.09 (522) | 58.62 (259) | 59.55 (263) |  |
| Other | 20.12 (178) | 21.22 (94) | 19.07 (84) |  |
| **Education status, % (n)** |  |  |  | 0.10 |
| Primary school graduate or below | 4.23 (37) | 4.60 (20) | 3.89 (17) |  |
| Middle/high/special school | 26.79 (237) | 29.81 (132) | 23.89 (105) |  |
| College graduate or above | 68.98 (610) | 65.60 (290) | 72.22 (320) |  |
| **PIR, % (n)** |  |  |  | 0.48 |
| low | 18.54 (164) | 18.94 (84) | 18.16 (80) |  |
| Moderate | 37.48 (331) | 38.98 (172) | 36.03 (159) |  |
| high | 43.98 (389) | 42.08 (194) | 45.81 (202) |  |
| **Physical activity** |  |  |  | 0.23 |
| Moderate, % (n) | 46.38 (410) | 46.34 (205) | 46.41 (205) |  |
| Vigorous, % (n) | 26.90 (238) | 27.76 (123) | 26.07 (115) |  |
| **Laboratory results** |  |  |  |  |
| TC, mmol/L | 4.88 (0.05) | 4.85 (0.08) | 4.90 (0.06) | 0.56 |
| TG, mg/dL | 1.33 (0.04) | 1.35 (0.07) | 1.31 (0.03) | 0.60 |
| HDL-C, mmol/L | 1.35 (0.02) | 1.33 (0.03) | 1.36 (0.02) | 0.43 |
| LDL-C, mg/dL | 2.92 (0.05) | 2.89 (0.07) | 2.94 (0.05) | 0.58 |
| Fasting glucose, mg/dL | 5.94 (0.08) | 5.89 (0.13) | 5.09 (0.07) | 0.57 |
| Cr, umol/L | 79.07 (0.81) | 80.54 (1.37) | 77.66 (0.98) | 0.11 |
| eGFR, ml/min/1.73m2 | 119.70 (1.70) | 119.77 (2.68) | 119.62 (2.10) | 0.97 |
| UA, umol/L | 333.84 (3.93) | 337.81 (6.45) | 330.03 (4.19) | 0.30 |
| HbA1c, % | 5.58 (0.04) | 5.58 (0.06) | 5.58 (0.04) | 0.97 |
| Selenium, umol/L | 1.68 (0.01) | 1.65 (0.02) | 1.70 (0.02) | 0.06 |
| Zinc, umol/L | 13.74 (0.12) | 13.36 (0.16) | 14.11 (0.18) | < 0.01 |
| **Disease** |  |  |  |  |
| Hypertension, % (n) | 45.77 (404) | 46.00 (203) | 45.55 (201) | 0.91 |
| Diabetes, % (n) | 14.52 (129) | 17.57 (78) | 11.60 (51) | 0.07 |
| Dyslipidemia, % (n) | 45.17 (399) | 45.07 (119) | 45.27 (200) | 0.97 |
| HF, % (n) | 2.55 (22) | 2.34 (10) | 2.75 (12) | 0.74 |
| CHD,% (n) | 3.11 (28) | 4.11 (18) | 2.15 (10) | 0.17 |
| Stroke,% (n) | 1.15 (10) | 1.46 (6) | 0.86 (4) | 0.45 |

Note: Data are expressed as mean (SE) and numbers (percentage) as appropriate. All estimates were weighted to be nationally representative.

Abbreviations: PIR: Ratio of family income to poverty; BMI: body mass index; DBP: diastolic blood pressure; SBP: systolic blood pressure; HbA1c: glycated hemoglobin; TG: triglycerides; TC: total cholesterol; LDL-C: lower-density lipoprotein cholesterol; HDL-C: high-density lipoprotein cholesterol; ALT: Alanine Aminotransferase; Cr: creatinine; UA: uric acid; eGFR: estimated glomerular filtration rate; HF: heart failure; CHD: coronary heart disease.

**Table S6** **The associations of serum copper with cardiovascular and all-cause mortality in the propensity score matched from the Nation Health and Nutrition Examination Survey 2011-2016**

| **Copper, μmol/L** | **Number of death/total** | **Crude Model**  **HR (95%CI)** | **P** | **Model I**  **HR (95%CI)** | **P** | **Model II**  **HR (95%CI)** | **P** |
| --- | --- | --- | --- | --- | --- | --- | --- |
| CVD mortality |  |  |  |  |  |  |  |
| Per 1 SD increase | 24/884 | 2.06 (1.54,2.76) | < 0.01 | 5.50 (2.87,10.55) | < 0.01 | 4.98 (2.25,11.00) | < 0.01 |
| Tertiles |  |  |  |  |  |  |  |
| T1 (<19.84) | 6/442 | Ref. | 1.0 | Ref. | 1.0 | Ref. | 1.0 |
| T2 (≥ 19.84) | 18/442 | 413 (1.06,16.01) | < 0.01 | 10.25 (2.14,49.08) | < 0.01 | 7.78 (1.35,44.93) | 0.02 |
| All-cause mortality |  |  |  |  |  |  |  |
| Per 1 SD increase | 95/884 | 1.71 (1.39,2.11) | < 0.01 | 2.57 (2.10,3.14) | < 0.01 | 2.42 (1.90,3.09) | < 0.01 |
| Tertiles |  |  |  |  |  |  |  |
| T1 (<19.84) | 26/442 | Ref. | 1.0 | Ref. | 1.0 | Ref. | 1.0 |
| T2 (≥ 19.84) | 69/442 | 3.38 (1.99,5.74) | < 0.01 | 3.72 (2.83,5.81) | < 0.01 | 3.91 (2.25,6.77) | < 0.01 |

Note: Crude Model: unadjusted any factor.

Model I: multi‐factor model adjusted for age, gender, marital, education, BMI, eGFR, HbA1c, and UA.

Model II: multi‐factor model adjusted for Model I, CHD, diabetes mellitus, hypertension, dyslipidemia, moderate PA, smoking status, and drinking status.

Abbreviations: 95% CI: 95% confidence interval; HR: hazard ratio; Ref: reference; BMI: body mass index; UA: uric acid; PA: Physical activity; CHD: coronary heart disease; HbA1c: glycated hemoglobin; CVD mortality: cardiovascular disease mortality; eGFR: estimated glomerular filtration rate.

**Table S7 Sensitive analysis of the association between the serum Cu and CVD and all-cause mortality in adult Americans based on the multiple-imputation analysis.**

| CVD mortality | | | | |
| --- | --- | --- | --- | --- |
| Dataset item | B | SE | HR (95%CI) | P |
| 1 | 0.86 | 0.26 | 2.37 (1.41, 3.97) | < 0.001 |
| 2 | 0.85 | 0.26 | 2.33 (1.40, 3.86) | < 0.001 |
| 3 | 0.85 | 0.26 | 2.38 (1.43, 3.96) | < 0.001 |
| 4 | 0.86 | 0.26 | 2.37 (1.43, 3.94) | < 0.001 |
| 5 | 0.87 | 0.26 | 2.38 (1.43, 3.95) | < 0.001 |
| Pooled estimates | 0.86 | 0.26 | 2.36 (1.42,3.92) | 0.001 |
| All-cause mortality |  |  |  |  |
| Dataset item | B | SE | HR (95%CI) | P |
| 1 | 0.52 | 0.11 | 1.68 (1.35,2.10) | < 0.001 |
| 2 | 0.53 | 0.11 | 1.70 (1.36,2.11) | < 0.001 |
| 3 | 0.53 | 0.11 | 1.68 (1.35,2.10) | < 0.001 |
| 4 | 0.52 | 0.11 | 1.68 (1.35,2.10) | < 0.001 |
| 5 | 0.52 | 0.11 | 1.68 (1.35,2.10) | < 0.001 |
| Pooled estimates | 0.52 | 0.11 | 1.69 (1.35,2.10) | < 0.001 |

Note: multiple-imputation methods are based on 5 replications and the Markov-chain Monte Carlo method in the SAS MI procedure. Item 0 is raw data from NHANES, and items 1-5 are replenished data from the SAS MI procedure that used 5 replications and the Markov-chain Monte Carlo method based on BMI, education, eGFR, drinking status, hypertension, and age. The 5 replenished data were used to explore the association between the serum Cu and CVD and all-cause mortality. The serum Cu was included as a continuous variable in the weighted Cox proportional hazards regression analysis, which was used to evaluate the association between the serum Cu and CVD and all-cause mortality, and the result was expressed as hazard ratios and 95% confidence intervals with the pre-defined model. The model was adjusted for age, gender, marital, education, BMI, eGFR, HbA1c, UA, CHD, diabetes mellitus, hypertension, dyslipidemia, moderate PA, smoking status, and drinking status. Then, pooled estimates from five imputed data.

Abbreviations: SE: Standard error; 95% CI: 95% confidence interval; HR: hazard ratio; Ref: reference; BMI: body mass index; UA: uric acid; PA: Physical activity; CHD: coronary heart disease; HbA1c: glycated hemoglobin; CVD mortality: cardiovascular disease mortality; eGFR: estimated glomerular filtration rate.

**Table S8. Main characteristics of the included studies in the meta‐analysis of serum Cu and CVD and all-cause mortality**

| References  (First author,  Year, Country/Region) | Source of  individuals | Outcomes | Study design | N | Measurement of Cu | Age  (year), Male (%) | HR (95%CI)  Highest vs lowest | Follow-up times  (years) | Adjusted covariates |
| --- | --- | --- | --- | --- | --- | --- | --- | --- | --- |
| Mamiemi 1998  , Finland | City of Turku | CVD mortality | Prospective Cohort | 344 | AAS | 65.0, 52.9% | 2.15 (1.32-3.48) # | 13 | Age, sex, smoking, alcohol, BMI, hypertension, diabetes, HDL-Cand triglycerides. |
| Shi 2021,  China | Dongfeng-Tongji (DF-TJ) cohort study | All-cause mortality,  CVD mortality | Prospective Cohort | 6155 | ICP-MS | 64.9, 50.1% | 1.73 (1.42–2.11) *  1.94 (1.45–2.58) # | 9.8 | Age, sex, BMI, smoking, drinking, education, PA, eGFR, hypertension, hyperlipidemia, diabetes, and future disease status. |
| Tonelli 2018,  Canada | Northern and Southern Alberta Renal Programs | All-cause mortality | Prospective Cohort | 1278 | ICP-MS | 62.0,61.4% | 3.68 (1.31 - 10.29) * | 2 | Age, sex, Ethnicity, BMI, AF, AMI, Malnutrition, Cancer, Cerebrovascular disease, CHF, Chronic lung disease, Diabetes mellitus, Dementia, Hypertension, Liver disease, PVD, Psychiatric disease, Substance misuse, Albumin, Creatinine. |
| Li 2023 , China  (present study) | NHANES | All-cause mortality,  CVD mortality | Prospective Cohort | 5412 | ICP-MS |  | 1.89 (1.35-2.61) *  1.87 (1.02-3.45) # |  | Age, gender, race, marital, education, BMI, waist circumference, eGFR, HLD-C, UA, diabetes mellitus, hypertension, dyslipidemia, moderate PA, smoking status, and drinking status. |

* all-cause mortality # CV mortality

LURIC study: Ludwigshafen Risk and Cardiovascular Health study; NHANES: National Health and Nutrition Examination Survey; AAS: atomic absorption spectrophotometer; ICP-M: Inductively coupled plasma mass spectroscopy; AF: atrial fibrillation; AMI: acute myocardial infarction; CHF: chronic heart failure; PVD: Peripheral vascular disease; CVD: cardiovascular disease. PA: physical activity; LDL-C: low-density lipoprotein cholesterol; HDL-C: high-density lipoprotein cholesterol; BMI: body mass index; SBP: systolic blood pressure; CRP: C-Reactive Protein; eGFR: estimated glomerular filtration rate; CAD: coronary atherosclerotic disease; STEMI: ST-elevation myocardial infarction; NSTEMI: Non-ST-elevation myocardial infarction; CHD: coronary heart disease; 95% CI: 95% confidence interval; BMI: body mass index; UA: uric acid; HR: hazard ratio.

**Table S9** **Quality assessment of included studies.**

| Author  (Publication Year) | Newcastle-Ottawa Scale | | | | | | | | | |
| --- | --- | --- | --- | --- | --- | --- | --- | --- | --- | --- |
|  | Selection | | | Comparability | | | Outcome | | | Total |
|  | a | b | c | d | e | f | g | h | i |  |
| Mamiemi 1998, Finland | 1 | 1 | 1 | 1 | 0 | 0 | 1 | 1 | 1 | 7 |
| Shi 2021, China | 1 | 1 | 1 | 1 | 0 | 0 | 1 | 1 | 1 | 7 |
| Tonelli 2018, Canada | 1 | 1 | 1 | 1 | 0 | 0 | 1 | 1 | 1 | 7 |
| Li 2023, China | 1 | 1 | 1 | 1 | 0 | 0 | 1 | 1 | 1 | 7 |

1. Representativeness of the exposed cohort.
2. Selection of the non-exposed cohort.
3. Ascertainment of exposure.
4. Demonstration that outcome of interest was not present at start of study.
5. Comparability of cohorts on the basis of the design or analysis (adjusted for age).
6. Comparability of cohorts on the basis of the design or analysis (adjusted for any other factor).
7. Assessment of the outcome.
8. Was follow-up long enough for outcomes to occur? (1 year for CVD and all-cause mortality).
9. Adequacy of follow-up of cohorts.

Li 2023, China: the present study.


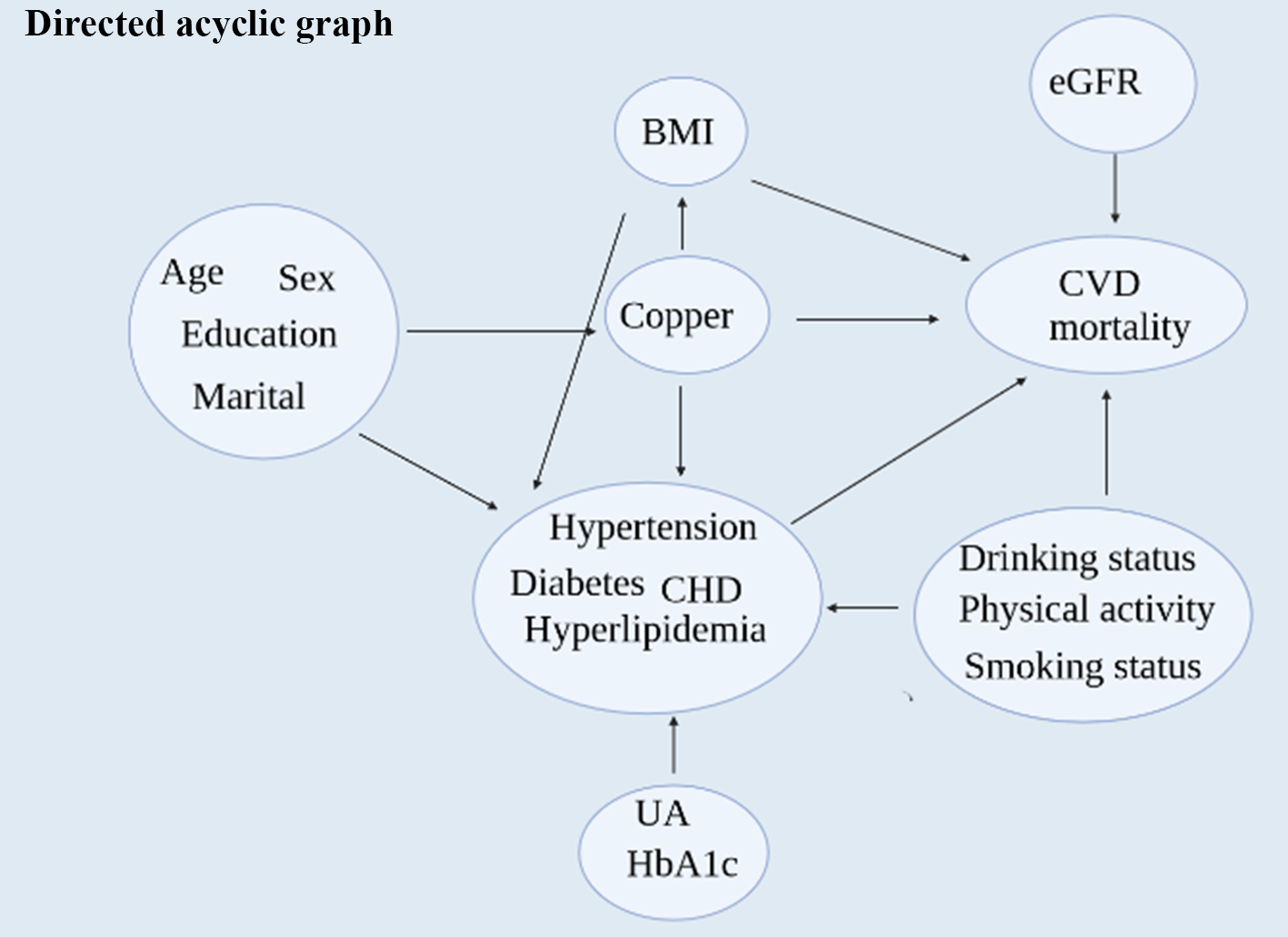


Abbreviations: BMI: body mass index; UA: uric acid; CHD: coronary heart disease; HbA1c: glycated hemoglobin; CVD mortality: cardiovascular disease mortality; eGFR: estimated glomerular filtration rate.

**Figure S1.** **Directed Acyclic Graph (DAG) of the association between serum copper and CVD and all-cause mortality.**


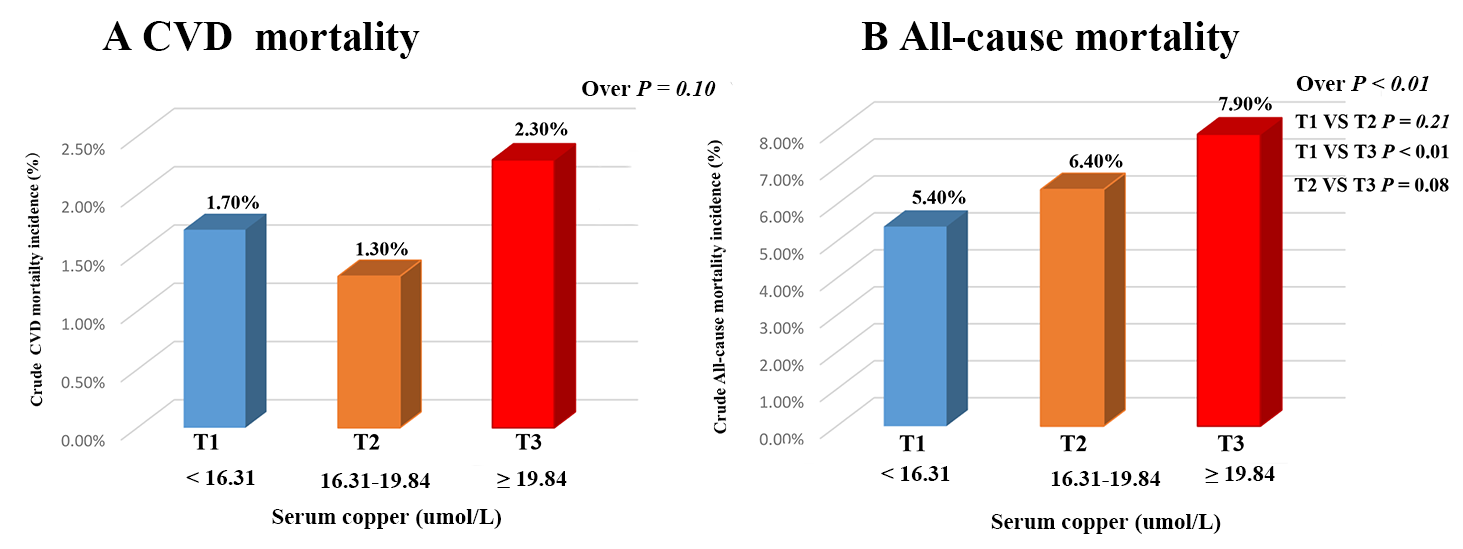


**Figure S2. Crude death rates according to tertiles of serum copper. (A) CVD death. (B) All-cause death.**

Note: Compared to the all-cause mortality of T1, the all-cause mortality of T2 was not significantly increased (P > 0.05), but T3 was higher in all-cause mortality (P < 0.05). The all-cause mortality of T3 was not significantly increased compared with T2 (P < 0.05).
